# Supplementary material for: Essential Oils as a Good Weapon against Drug-Resistant Candida auris
Source: Antibiotics (Basel). 2022 Jul 20;11(7):977. doi: 10.3390/antibiotics11070977 (PMC9311903; doi:10.3390/antibiotics11070977)
Supplement: Supplementary file 1 [file antibiotics-11-00977-s001.zip › antibiotics-1776780-supplementary.pdf]

Supplementary Material

1. **Adaptation of the Essential Oil Analysis Bulletins provided by the company florame® (Saint-Rémy-de-Provence, France).**

1.1 Tea Tree Essential Oil Analysis Report Lot 903025

Identification:

- **Name (International Nomenclature of Cosmetic Ingredients (INCI)/CTFA):** *Melaleuca alternifolia* (Tea tree) leaf oil
- **Latin name:** *Melaleuca alternifolia*
- **N° CAS:** 85085-48-9
- **N° EINECS (European Inventory of Existing Commercial Chemical Substances):** 285-377-1

General characteristics:

- **Botanical family:** Myrtaceae
- **Product acquisition:** Steam distillation of leaves and terminal branches
- **Main molecules :** Terpinen-4-ol, γ-terpinene, α-terpinene
- **Shelf life:** Contains no preservatives, can be stored in its original state away from light, air and heat according to the best before end (BBE).

Lot characteristics:

- **Origin:** South Africa
- **Quality:** Organic (certified by Ecocert SAS F32600)
- **Texture / Color / Odor:** Clear fluid liquid / colorless / characteristic
- **Refractive index (20°C):** 1.4775
- **Density (20°C):** 0.893
- **Rotary power (20°C):** 9.9°

Lot information:

- **Distillation date:** 12/2018
- **BBE:** 12/2023

Chromatographic analysis conditions:

- **Device:** GC with dual FID detection
- **Carrier gas:** Hydrogen
- **Polar column:** Elite Vax (100% polyethylene glycol) 60 m / 0.25 mm / 0.25 µm
- **Nonpolar Column:** Elite 5 (5% diphenyl 95% dimethylpolysiloxane) 60 m/0.25 mm/0.25 µm

Analysis date: 03/09/2019

<u>Chromatographic profiles:</u>

- **Polar column analysis results**

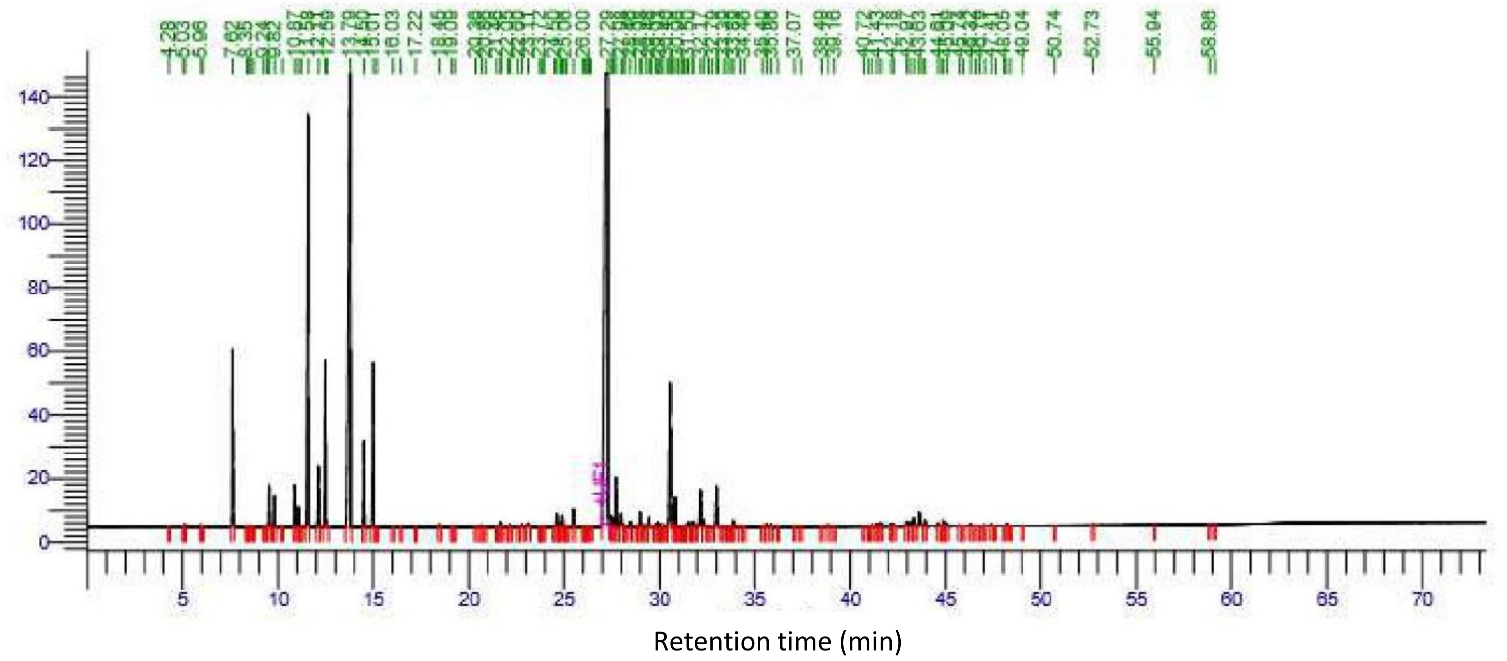

Order peaks	Molecules name	Retention time (min)	Area (uV*sec)	Area (%)
6	ALPHA THUYENE+ALPHA PINENE	7.621	174708.63	3.23
13	BETA PINENE	9.537	35106.71	0.65
14	MYRCENE	9.816	25279.06	0.47
16	ALPHA PHELLANDRENE	10.874	38765.73	0.72
17		11.063	18977.32	0.35
19	ALPHA TERPINENE	11.580	554375.94	10.24
20	LIMONENE	12.108	56530.77	1.04
21	1.8 cineole + beta phellandren	12.496	199528.39	3.68
23	GAMMA TERPINENE	13.790	1171977.05	21.64
24	PARA CYMENE	14.505	78064.17	1.44
25	TERPINOLENE	15.011	182273.45	3.37
37		21.650	4934.52	0.09
43		23.110	4023.26	0.07

Order peaks	Molecules name	Retention time (min)	Area (uV*sec)	Area (%)
48	ALPHA GURGUJENE	24.626	13106.23	0.24
49		24.798	2908.32	0.05
50		24.914	12694.02	0.23
53	LINALOL	25.510	19292.58	0.36
59	TERPINENE-4-OL	27.292	2304102.68	42.54
60		27.445	9472.94	0.17
61		27.578	8815.55	0.16
62	TRANS BETA CARYOPHYLLENE	27.720	47390.94	0.87
63		27.982	18393.72	0.34
66		28.456	5128.79	0.09
68	AROMADENDRENE	28.978	16387.62	0.30
69		29.064	3199.68	0.06
71	ALLOAROMADENDRENE	29.464	10698.07	0.20
73		29.818	2822.20	0.05
74		29.932	4384.49	0.08
78	ALPHA TERPINEOL	30.567	175826.61	3.25
79		30.660	7470.76	0.14
80	LEDENE	30.812	34932.93	0.64
85		31.498	4692.54	0.09
86		31.687	4870.87	0.09
87		31.799	8641.04	0.16
88		32.167	40120.63	0.74
89	B SELINENE	32.322	7412.44	0.14
92	DELTA CADINENE	33.007	46889.96	0.87
97		33.886	6256.89	0.12
101		35.628	2582.23	0.05
113		41.623	3409.67	0.06
116		42.971	5437.12	0.10
117		43.126	4489.82	0.08
118		43.345	8506.54	0.16
119	GLOBULOL	43.629	15393.76	0.28
121	VIRIDIFLOROL	43.928	7175.49	0.13
122		44.614	3101.44	0.06
124		44.903	5668.28	0.10
		1288.910	5416221.91	100.00

- **Nonpolar column analysis results**

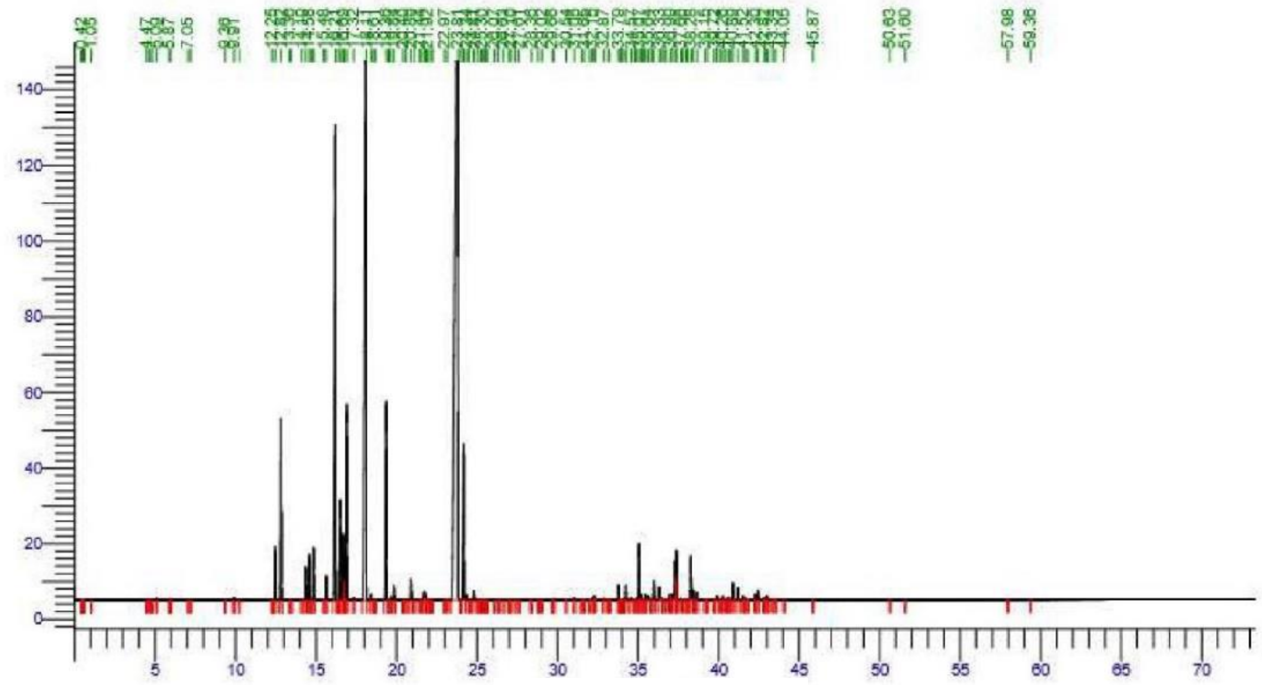

Order peaks	Molecules name	Retention time (min)	Area (uV*sec)	Area (%)
18	ALPHA THUYENE	12.457	38299.14	0.71
19	ALPHA PINENE	12.819	135751.17	2.52
23	SABINENE	14.344	25385.25	0.47
24	BETA PINENE	14.578	35441.41	0.66
26	MYRCENE	14.856	39017.45	0.72
28	ALPHA PHELLANDRENE	15.621	19700.14	0.37
29	ALPHA TERPINENE	16.206	548966.31	10.19
31	PARA CYMENE	16.493	80344.96	1.49
32	LIMONENE	16.688	58398.53	1.08
33	BETA PHELLANDRENE	16.763	36761.26	0.68
34	1,8-CINEOLE	16.904	163213.77	3.03
35		17.324	2588.68	0.05
36	GAMMA TERPINENE	18.108	1151919.23	21.38
37		18.424	5192.96	0.10
40	TERPINOLENE	19.365	186181.18	3.46
42		19.657	2961.01	0.05
43		19.837	12369.57	0.23
46	LINALOL	20.891	19478.89	0.36
50		21.741	13795.86	0.26
51		21.829	3605.85	0.07
57	TERPINEN-4-OL	23.810	2273456.35	42.20
59	ALPHA TERPINEOL	24.176	174851.30	3.25
60	ALPHA COPAENE	24.343	5309.95	0.10
62		24.813	7490.93	0.14
87		32.286	3013.81	0.06
92	ALPHA GURJUNENE	33.788	13362.52	0.25
96	TRANS BETA CARYOPHYLLENE	34.249	13469.54	0.25
100	AROMADENDRENE	35.069	53710.14	1.00
101		35.192	4590.32	0.09
103		35.451	4960.53	0.09
104		35.645	4202.51	0.08
106	ALLOAROMADENDRENE	35.970	17044.86	0.32
107		36.333	12709.77	0.24
111		36.990	4829.62	0.09
112		37.085	8570.32	0.16
113	LEDENE	37.302	44215.27	0.82
114	BICYCLOGERMACRENE	37.388	45525.47	0.85
119	DELTA CADINENE	38.276	42044.94	0.78
120		38.422	8865.37	0.16
121		38.681	6841.61	0.13
125		39.902	4160.48	0.08
127		40.259	3170.96	0.06
131	GLOBULOL	40.887	15687.58	0.29
132	VIRIDIFLOROL	41.218	13036.92	0.24
133		41.525	2967.01	0.06
136		42.296	5763.16	0.11
137		42.444	8740.30	0.16
139		42.977	4836.16	0.09
		1351.683	5386800.33	100.00

1.2 Cajeput Essential Oil Analysis Report Lot 801736

Identification:

- **Name (INCI/CTFA):** *Melaleuca leucadendra* Cajeput oil
- **Latin name:** *Melaleuca cajaputi*
- **N° CAS :** 85480-37-1
- **N° EINECS:** 287-316-4

General characteristics:

- **Botanical family:** Myrtaceae
- **Product acquisition:** Steam distillation of organic cajeput leaves
- **Main molecules:** 1,8-cineole, α-terpineol, Terpinolene
- **Allergens:** Limonene, linalool, geraniol
- **Shelf life:** Contains no preservatives, can be stored in its original state away from light, air and heat according to the BBE. (5 years from the date of distillation)

Lot characteristics:

- **Origin:** South Africa
- **Quality:** Organic (certified by Ecocert SAS F32600)
- **Texture / Color / Odor:** Clear liquid/ pale yellow/ nice fresh
- **Refractive index (20°C):** 1.4676
- **Density (20°C):** 0,914
- **Rotary power (20°C):** -1.2°

Lot information:

- **Distillation date:** 09/2017
- **BBE:** 09/2022

Chromatographic analysis conditions:

- **Device:** GC with dual FID detection
- **Carrier gas:** Hydrogen
- **Polar column:** Elite Vax (100% polyethylene glycol) 60 m / 0.25 mm / 0.25 µm
- **Nonpolar Column:** Elite 5 (5% diphenyl 95% dimethylpolysiloxane) 60 m/0.25 mm/0.25 µm

Analysis date: 12/02/2019

<u>Chromatographic profiles:</u>

- **Polar column analysis results**

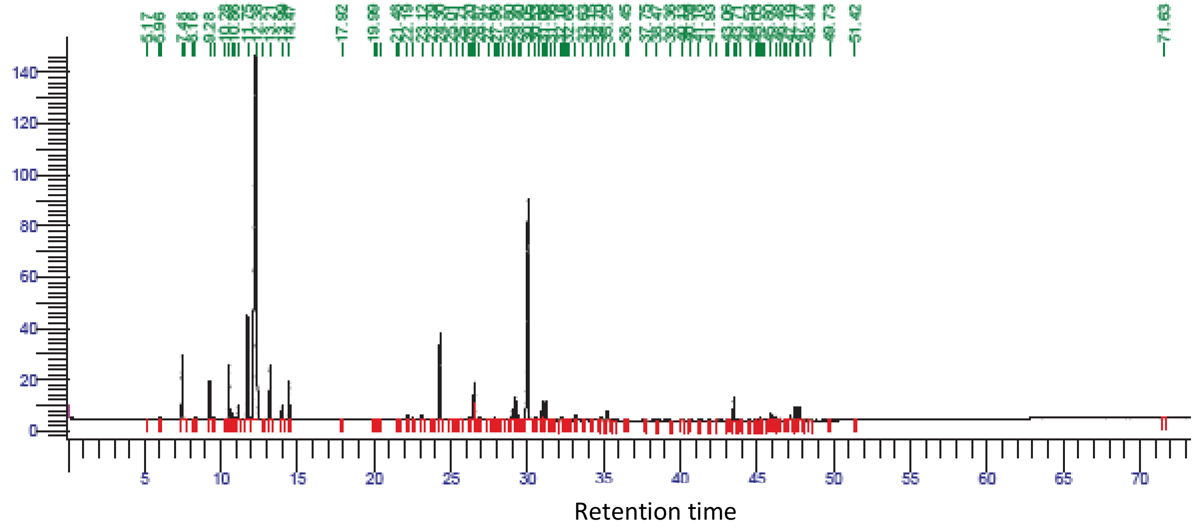

Order peaks	Molecules name	Retention time (min)	Area (uV*sec)	Area (%)
4	ALPHA PINENE+ALPHA THUYENE	7.478	78610.30	1.90
8	BETA PINENE	9.278	50998.47	1.23
11	MYRCENE	10.549	74151.75	1.79
12	ALPHA PHELLANDRENE	10.728	8094.94	0.20
13		10.857	2696.55	0.07
14	ALPHA TERPINENE	11.149	22526.28	0.54
15	LIMONENE	11.753	214433.02	5.18
16	1.8CINEOLE	12.380	2485907.17	60.01
18	Trans Beta Ocimene	13.214	69722.17	1.68
19	PARA CYMENE	13.990	18346.58	0.44
20	TERPINOLENE	14.467	51574.62	1.24
27		22.187	7727.52	0.19
28	ALPHA COPAENE	22.533	2624.30	0.06
29		23.116	6308.71	0.15
32	LINALOL	24.301	133142.59	3.21
35		25.711	3083.45	0.07
36		26.197	2571.83	0.06
37	TERPINEN-4-OL	26.273	2832.99	0.07
38	TRANS BETA CARYOPHYLLENE	26.469	38090.64	0.92
39		26.559	58194.41	1.40
40		26.867	3627.97	0.09
42		27.855	2815.07	0.07
47		29.031	18098.05	0.44
48	ALPHA CARYOPHYLLENE+	29.215	40929.65	0.99
49		29.401	7284.38	0.18
52	alpha terpineol	30.050	491423.03	11.86

Order peaks	Molecules name	Retention time (min)	Area (uV*se	Area (%)
53		30.529	3938.34	0.10
54		30.699	2672.43	0.06
55	GERMACRENE D	30.925	15056.54	0.36
56	BETA SELINENE	31.058	33896.43	0.82
57	ALPHA SELINENE	31.228	36747.36	0.89
61		32.160	3788.04	0.09
62		32.314	3593.15	0.09
66	DELTA CADINENE	33.135	10199.93	0.25
70	NERAL	34.869	6529.47	0.16
71	GERANIOL	35.253	14932.84	0.36
85	OXYDE DE CARYOPHYLLENE	43.491	36465.43	0.88
91		45.215	3750.76	0.09
92		45.353	3092.93	0.07
93		45.449	2623.26	0.06
95		45.917	10618.04	0.26
96	10 EPI GAMMA EUDESMOL	46.195	6218.52	0.15
100		47.188	8246.28	0.20
101	ALPHA EUDESMOL	47.496	20335.64	0.49
102	BETA EUDESMOL	47.767	21362.82	0.52
104		48.445	2882.60	0.07
		1306.294	4142767.25	100.00

- **Nonpolar column analysis results**

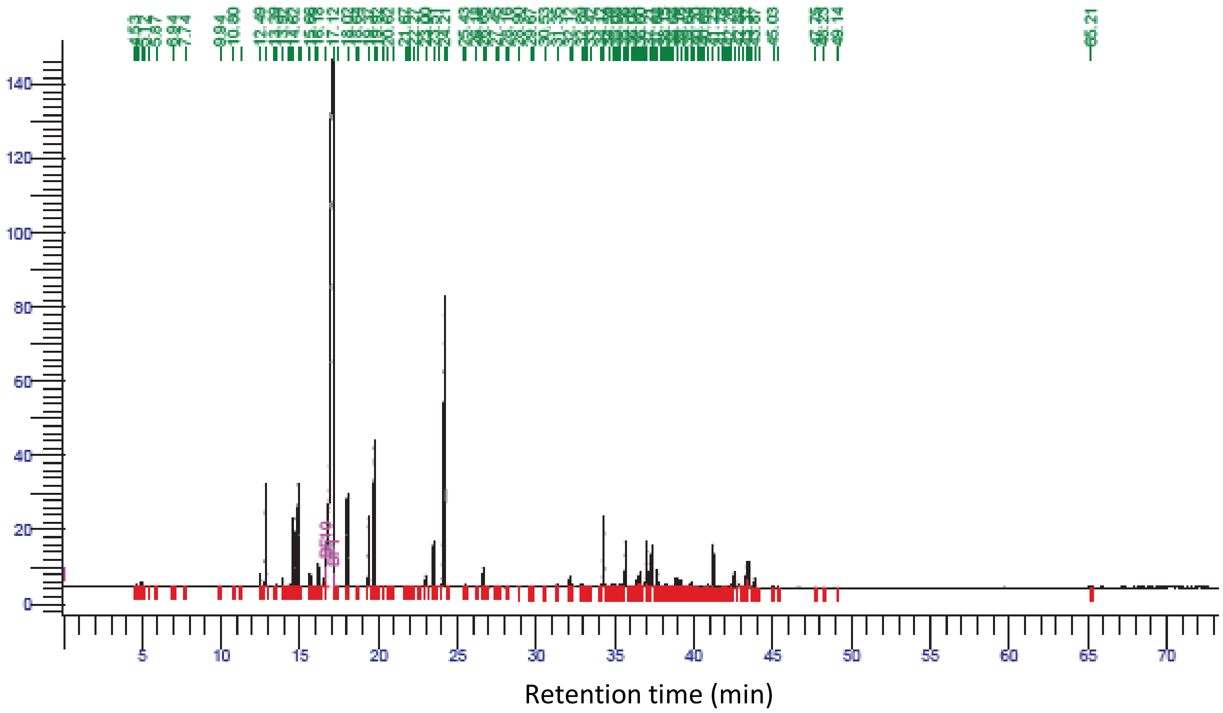

Order peaks	Molecules name	Retention time (min)	Area (uV*sec)	Area (%)
14	ALPHA THUYENE	12.487	9033.82	0.20
15	ALPHA PINENE	12.846	76696.78	1.71
18	BENZALDEHYDE	13.924	7044.73	0.16
21	SABINENE	14.618	55361.99	1.23
22	MYRCENE	14.900	81407.53	1.81
24	ALPHA PHELLANDRENE	15.657	11717.37	0.26
26	ALPHA TERPINENE	16.180	24474.28	0.54
27	PARA CYMENE	16.597	19413.07	0.43
28	CINEOLE	17.124	2916315.41	64.83
30	GAMMA TERPINENE	18.022	76858.90	1.71
33	TERPINOLENE	19.368	58775.31	1.31
34	LINALOL	19.737	147078.71	3.27
44	BORNEOL	22.999	11096.17	0.25
45	TERPINEN-4-OL	23.513	41001.50	0.91
47	ALPHA TERPINEOL	24.214	503307.28	11.19
52		26.649	16114.28	0.36
62		31.347	2917.49	0.06
63	ACETATE DE GERANYLE	32.122	11159.22	0.25
65	ALPHA COPAENE	32.887	2836.07	0.06
70		34.280	66868.64	1.49
72		34.853	2884.59	0.06
75		35.350	4473.88	0.10
76		35.564	3046.56	0.07
77	ALPHA CARYOPHYLLENE	35.673	41300.17	0.92
83		36.430	7138.92	0.16
84		36.511	9610.44	0.21
85	D GERMACRENE	36.602	14717.22	0.33
87		36.917	4758.68	0.11
88	BETA SELINENE	37.010	57450.39	1.28
89		37.207	4083.18	0.09
90	ALPHA SELINENE	37.336	40054.39	0.89
93		37.678	17929.24	0.40
97		38.258	3407.42	0.08
102		38.902	7708.87	0.17
103		39.160	6670.84	0.15
107		39.828	3944.15	0.09
115	OXYDE DE CARYOPHYLLENE	41.231	39900.20	0.89
123	10 EPI GAMMA EUDESMOL	42.345	4291.27	0.10
124	ALPHA EUDESMOL	42.580	24934.28	0.55
127		43.369	25386.05	0.56
128	BETA EUDESMOL	43.458	25799.75	0.57
130		43.869	9213.11	0.20
		1269.604	4498182.15	100.00

1.3 Niaouli Essential Oil Analysis Report Lot 100378

Identification:

- **Name (INCI/CTFA):** *Melaleuca viridiflora* leaf oil
- **Latin name:** *Melaleuca quinquenervia*
- **N° CAS:** 132940-73-9
- **N° EINECS:** 310-217-5

General characteristics:

- **Botanical family:** Myrtaceae
- **Product acquisition:** Steam distillation of leaves
- **Main molecules:** 1,8-cineole, Viridiflorol
- **Allergens:** Limonene, linalool, Citronellol
- **Shelf life:** Contains no preservatives, can be kept as is for 5 years away from light, air and heat.

Lot characteristics:

- **Origin:** Madagascar
- **Quality:** Organic (certified by Ecocert SAS F32600)
- **Texture / Color / Odor:** Clear fluid liquid / colorless / characteristic
- **Refractive index (20°C):** 1,464
- **Density (20°C):** 0,910

Lot information:

- **Distillation date:** 09/2017
- **BBE:** 09/2022

Chromatographic analysis conditions:

- **Device:** GC 6890 MS 5975
- **Mass range:** 40-450 uma
- **Column:** HP5ms/ 30 m/ 0.25 mm/ 0.25 µm
- **Injection divided:** 1 EL - ratio 1/200
- **Carrier gas:** Helium / Flow rate: 1 mL/min
- **Oven:** 2°/min, 60-250°C for 5 min
- **Acquisition time:** 100 min

Analysis date: 14/03/11

Chromatographic profiles:

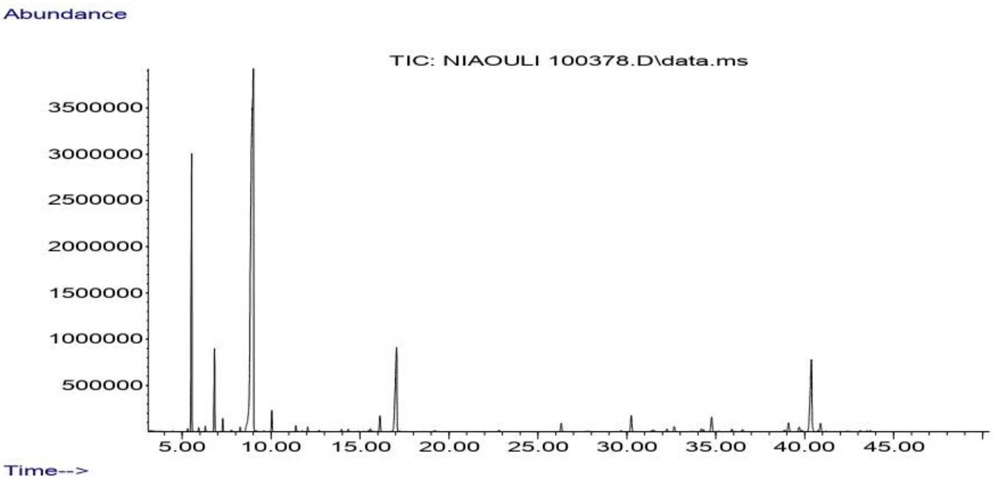

Identification	Area (%)
α-Thuyène	0,11
α-Pinène	12,00
Camphène	0,18
Benzaldéhyde	0,22
β-Pinène	3,46
β–Myrcène	0,56
α-Phellandrène	0,11
α-Terpinène	0,26
p-Cymène	57,14
**Limonène***	
1,8-Cinéole	
trans-β-Ocimène	0,04
γ-Terpinène	1,04
Terpinolène	0,34
Benzoate de méthyle	0,05
**Linalol***	0,24
Alcool fenchylique	0,07
Isopulegol	0,13
Bornéol	0,06
Terpinèn-4-ol	1,01
α-Terpinéol	8,55
**Citronellol***	0,07
Patchoulène	0,06
β-Caryophyllène	1,22
α-Caryophyllène	0,20
Alloaromadendrène	0,38
β-Sélinène	0,20
Lédène	1,19
γ-Cadinène	0,18
δ-Cadinène	0,16
Nérolidol	0,73
Viridiflorol	7,29
Ledol	0,73

1.4 White thyme Essential Oil Analysis Report Lot 800180

Identification:

- **Name (INCI/CTFA):** *Thymus satureiodes* (White thyme) oil
- **Latin name:** *Thymus satureiodes*

General characteristics:

- **Botanical family:** Lamiaceae
- **Product acquisition:** Steam distillation of flowered tops
- **Main molecules:** Borneol, thymol, carvacrol
- **Allergens:** Limonene, linalool
- **Shelf life:** Contains no preservatives, can be stored in its original state away from light, air and heat according to the BBE. (5 years from the date of distillation)

Lot characteristics:

- **Origin:** Morocco
- **Quality:** Organic (certified by Ecocert SAS F32600)
- **Texture / Color / Odor:** Clear liquid/light yellow/characteristic
- **Refractive index (20°C):** 1,4843
- **Density (20°C):** 0,938
- **Rotary power (20°C):** -6.0°

Lot information:

- **Distillation date:** 06/2016
- **BBE:** 06/2022

Chromatographic analysis conditions:

- **Device:** GC with dual FID detection
- **Carrier gas:** Hydrogen
- **Polar column:** Elite Vax (100% polyethylene glycol) 60 m / 0.25 mm / 0.25 µm
- **Nonpolar Column:** Elite 5 (5% diphenyl 95% dimethylpolysiloxane) 60 m/0.25 mm/0.25 µm

Analysis date: 19/01/2018

<u>Chromatographic profiles:</u>

- **Polar column analysis results**

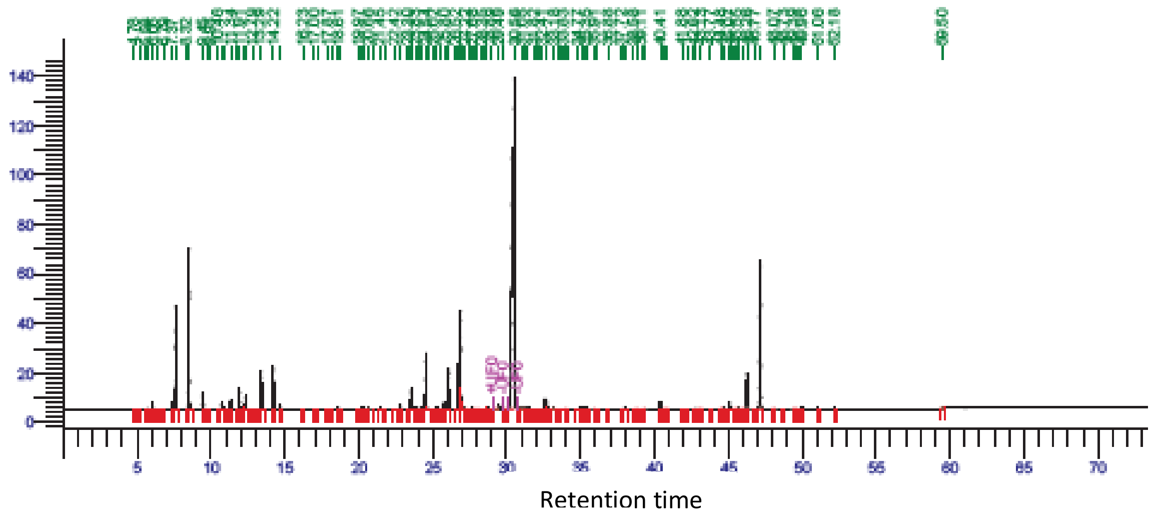

Order peaks	Molecules name	Retention time (min)	Area (uV*sec)	Area (%)
6	ETHANOL	6.054	7094.70	0.24
9	TRICYCLENE	7.369	9965.88	0.34
10	ALPHA PINENE + alpha thuyene	7.616	128746.13	4.41
12	CAMPHENE	8.516	210892.53	7.22
13	BETA PINENE	9.448	23931.73	0.82
18	MYRCENE	10.726	10753.14	0.37
19		10.911	2647.77	0.09
20	ALPHA TERPINENE	11.338	14926.23	0.51
22	LIMONENE	11.914	31283.70	1.07
23		12.240	5574.19	0.19
24	1.8 CINEOLE	12.336	22850.53	0.78
28	GAMMA TERPINENE	13.406	55362.02	1.89
29	PARA CYMENE	14.219	62950.12	2.15
30	TERPINOLENE	14.682	8260.80	0.28
40		20.259	3274.99	0.11
44		21.448	2705.77	0.09
47		22.801	6685.40	0.23
50	CAMPHRE	23.541	37470.10	1.28
53		24.265	2864.65	0.10
54	LINALOL	24.537	86627.94	2.97
57		25.258	3590.73	0.12
59		25.798	12035.88	0.41
60	TERPINEN-4-OL	26.075	72461.93	2.48
62	ACETATE BORNYLE	26.736	84606.05	2.90
63	BETA CARYOPHYLLENE	26.884	192970.47	6.61
64		26.982	10848.40	0.37
67		27.684	5298.29	0.18

Order peaks	Molecules name	Retention time (min)	Area (uV*sec	Area (%)
72		28.622	3471.21	0.12
73		28.673	4397.72	0.15
75		29.487	12665.27	0.43
77	ALPHA TERPINEOL + borneol	30.557	1377321.51	47.14
78		31.047	5737.33	0.20
79		31.216	7760.70	0.27
80		31.452	3305.62	0.11
85	DELTA CADINENE	32.602	16630.66	0.57
86	GAMMA CADINENE	32.717	14615.49	0.50
87		33.183	2886.21	0.10
95		35.180	3397.70	0.12
97		35.455	4032.47	0.14
103		38.027	2552.99	0.09
108	OXYDE DE CARYOPHYLLENE	40.409	11249.26	0.39
121		45.065	11614.11	0.40
127	THYMOL METHYL ETHER+THYMOL	46.264	66214.83	2.27
129	CARVACROL	47.155	251009.52	8.59
135		49.960	2748.62	0.09
138		59.499	3254.96	0.11
		1179.615	2921546.24	100.00

- **Nonpolar column analysis results**

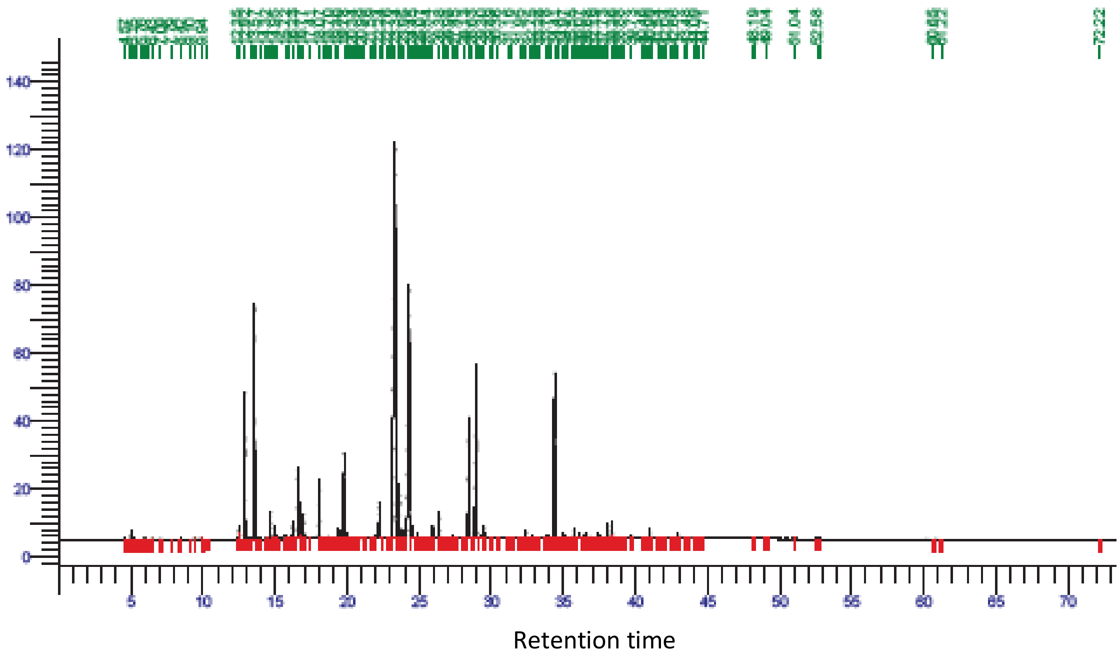

Order peaks	Molecules name	Retention time (min)	Area (uV*sec)	Area (%)
3	ETHANOL	5.014	5059.16	0.17
21	TRICYCLENE	12.492	9749.14	0.33
22	ALPHA THUYENE	12.550	7304.25	0.25
24	ALPHA PINENE	12.915	120496.01	4.12
26	CAMPHENE	13.574	212341.40	7.26
31	BETA PINENE	14.680	22948.70	0.78
33	MYRCENE	14.949	10346.93	0.35
34		15.105	2665.07	0.09
36		15.719	3331.22	0.11
38	ALPHA TERPINENE	16.229	14216.65	0.49
39	PARA CYMENE	16.580	62538.70	2.14
40	LIMONENE	16.771	31166.63	1.07
41		16.847	3899.89	0.13
42	1.8 CINEOLE	16.975	21380.12	0.73

Order peaks	Molecules name	Retention time (min)	Area (uV*sec)	Area (%)
44	GAMMA TERPINENE	18.070	53448.21	1.83
51	TERPINOLENE	19.424	12054.45	0.41
52	LINALOL	19.784	85262.77	2.92
53		19.982	6941.29	0.24
64		21.930	3976.25	0.14
65	CAMPHRE	22.242	37158.00	1.27
70	BORNEOL	23.339	932162.15	31.88
71	TERPINEN-4-OL	23.639	54082.31	1.85
73		23.951	12391.28	0.42
74	ALPHA TERPINEOL	24.308	466443.32	15.95
76		24.527	11005.95	0.38
78		24.893	4514.00	0.15
85		25.939	11722.05	0.40
86	THYMOL METHYL ETHER	26.378	24022.21	0.82
90		27.311	3768.42	0.13
94	ACETATE BORNYLE + THYMOL	28.431	132755.89	4.54
96	CARVACROL	28.975	252264.74	8.63
98		29.297	4294.88	0.15
99		29.497	11246.06	0.38
109		32.398	6204.34	0.21
116		33.896	2856.04	0.10
118	TRANS BETA CARYOPHYLLENE	34.413	193689.83	6.62
120		34.972	5691.97	0.19
124		35.757	9420.71	0.32
126		36.074	4891.25	0.17
129		36.532	4701.20	0.16
136		37.455	4538.33	0.16
140	GAMMA CADINENE	38.081	13674.37	0.47
141	DELTA CADINENE	38.355	13002.49	0.44
151	OXYDE DE CARYOPHYLLENE	40.986	10665.14	0.36
161		42.936	7401.58	0.25
		1104.173	2923755.33	100.00
